# Characterization and Antioxidant Activities of Yellow Strain *Flammulina velutipes* (Jinhua Mushroom) Polysaccharides and Their Effects on ROS Content in L929 Cell

**DOI:** 10.3390/antiox8080298

**Published:** 2019-08-10

**Authors:** Yu-Ning Hu, Tzu-Jung Sung, Chun-Hsu Chou, Kai-Lun Liu, Liang-Po Hsieh, Chang-Wei Hsieh

**Affiliations:** 1 Department of Food Science and Biotechnology, National Chung Hsing University, 145 Xingda Rd., South Dist., Taichung City 402, Taiwan; 2 DR JOU BIOTECH CO., LTD, No.21, Lugong S. 2nd Rd., Lukang Township, Changhua Country 505, Taiwan; 3 Department of Neurology, Cheng Ching General Hospital, Taichung 404, Taiwan; 4 Department of Medical Research, China Medical University Hospital, Taichung 404, Taiwan

**Keywords:** *Flammulina velutipes*, polysaccharide, antioxidant activities, chemical composition, fractional precipitation, ROS production

## Abstract

Yellow strain *Flammulina velutipes*, which is known as Jinhua mushroom in Taiwan, has become popular among customers due to its distinct texture that is utterly different from white strain *F. velutipes*. However, there has been little study on the physicochemical properties, antioxidant activities, and biological functions of yellow strain *F. velutipes* polysaccharides (FVYs). The specific aims of this study are to evaluate and compare the physicochemical properties, antioxidant activities, and biological functions of FVYs and white strain *F. velutipes* polysaccharides (FVWs) in order to select the strain appropriate for cosmetic ingredient. The FVYs and FVWs were prepared by fractional precipitation (40%, 60%, and 80%). According to the results, FVY-80 showed the greatest antioxidant activities based on 2,2-diphenyl-1-picrylhydrazyl (DPPH) (IC_50_ = 2.22 mg/mL) and 2,2’ -azino-bis-3-ethylbenzthiazoline-6-sulphonic acid (ABTS) radical assay (IC_50_ = 2.04 mg/mL). None of the fractions exhibited cytotoxicity toward L929 cell under a concentration of 500 μg/mL. FVY-80 significantly reduced the reactive oxygen species (ROS) content in L929 cell by 55.96%, as compared with the H_2_O_2_-induced L929 cell, according to the dichloro-dihydro-fluorescein diacetate (DCFH-DA) assay. In conclusion, we suggest that FVY-80 is the best source for a cosmetics ingredient.

## 1. Introduction

**Figure 1:**
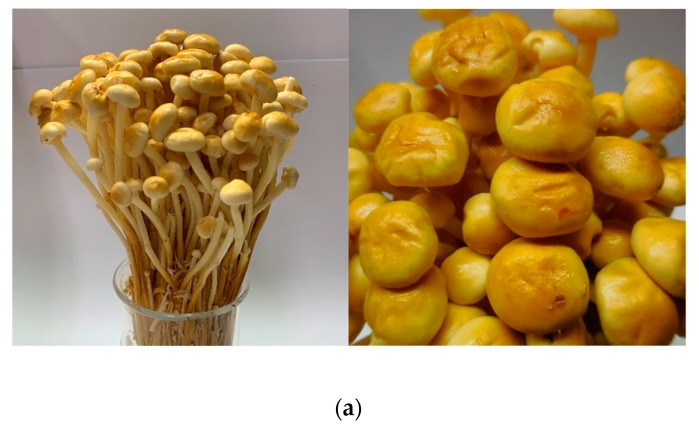

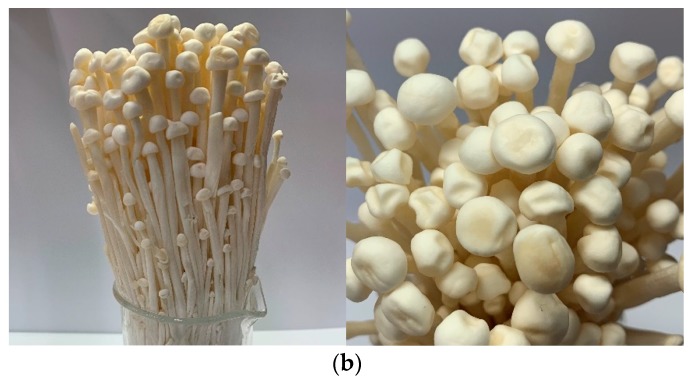
Two strains of *F. velutipes*: (**a**) yellow strain; and, (**b**) white strain.

*Flammulina velutipes* (*F. velutipes*), which is a strain of the edible mushrooms, contains high amounts of essential amino acids, dietary fiber, polysaccharides, and steroids. Its nutritional properties and attractive flavor have made it popular in Japan and China [1,2,3]. *F. velutipes* has two different strains that are distinguished by their stipe color. One is white stipe, known as “Jinzhen mushroom” and it originated from Japan, and is widely known [4]. The other one is the yellow strain, whose cap has sticky fluid, and it is called “Jinhua mushroom” in Taiwan (Figure 1). The yellow strain *F. velutipes* has become more acceptable to consumers in recent years due to its distinct texture and unique flavor that are utterly different from white strain *F. velutipes.* In Taiwan, a majority of mushroom cultivators in Taiwan prefer to cultivate yellow strain *F. velutipes* rather than white strain *F. velutipes,* because they believe that yellow strain *F. velutipes* is more valuable than white strain *F. velutipes* in both nutrition and applications. However, little scientific evidence has been provided on the values of yellow strain *F. velutipes*. Therefore, it is important to scientifically prove which mushroom strain has more health benefits and is more worthy of mass cultivation.

Reactive oxygen species (ROS) has been recently emphasized, since excessive ROS can damage cells, lipids, proteins, and DNA, thereby leading a variety of diseases, including inflammation, atherosclerosis, aging, diabetes, and cancer [5,6]. New interest has arisen in utilizing antioxidants from natural sources in food, cosmetic, and pharmaceutical industries, owing to the worries of adverse effects of synthetic antioxidants. Besides, excessive ROS would upregulate matrix metallopeptidases (MMPs) expression and downregulate collagen synthesis genes in dermal fibroblast, damaging skin integrity, and finally leading to old-looking skin, such as thin, fragile, and wrinkled skin, and age-related skin disorders, such as impaired wound healing and skin cancer development. Treatment with antioxidants is an effective approach to defending oxidative stress and improving skin aging and wound healing [7,8]. Therefore, L929 cells are widely utilized to assess the antioxidant activity of natural antioxidant *in vitro* [9].

Glycoprotein and polysaccharide-protein complexes from fungi have been identified as the main active substances [10]. According to the previous studies, *Trametes versicolor* polysaccharopeptides (TVPs) that were obtained from the enzymatic hydrolysis process showed that the biological activities are associated to molecular weight distribution; the lower molecular weight TVPs has better free radical scavenging ability than the higher molecular weight TVPs [11]. *Pholiota nameko* polysaccharide, as purified by fractional precipitation, demonstrated that the antioxidant activity of polysaccharides was determined by chemical composition, sugar unit, glycosidic linkage, molecular weight, degree of branching, and configuration, which suggested that antioxidant capacity of polysaccharide was influenced by multiple factors [1,12]. It was indicated that white strain *F. velutipes* polysaccharide has antitumor, memory and learning improvement, antioxidant activity, immunomodulatory properties, and hepatoprotective activity [1,3,13,14,15]. In addition, white strain *F. velutipes* SF-06 exopolysaccharides have considerable antioxidant capacity based on reducing hydroxyl radical, superoxide anion radical, reducing power and DPPH scavenging assay, and have anti-aging activation in aging mice through up-regulating catalase and total antioxidant capacity and down-regulating the malondialdehyde (MDA) content [16]. However, numerous studies reported the physicochemical, antioxidant, and biological properties of white strain *F. velutipes* polysaccharides, little is known about those of the yellow strain *F. velutipes* polysaccharides; moreover, their potential biological effects on the cosmetic applications have yet to be discussed.

In this work, the dried fruiting body of yellow strain *F. velutipes* was purified by fractional precipitation, an effective way for obtaining the different-feature polysaccharides [17], while using the final concentration of alcohol as 40%, 60%, and 80%, respectively. We aim to characterize yellow strain *F. velutipes* polysaccharides (FVYs) and white strain *F. velutipes* polysaccharides (FVWs) while using constituent, monosaccharide component, and FT-IR spectroscopic analysis, and evaluated their antioxidant activities using DPPH and ABTS radical scavenging assay, and their effects against H_2_O_2_-induced oxidative stress in L929 cells using dichloro-dihydro-fluorescein diacetate (DCFH-DA) assay in order to understand which strain is more appropriate for cosmetic applications.

## 2. Materials and Methods

### 2.1. Materials and Chemicals

Two strains of *F. velutipes* that were cultivated in Nantou County (Taiwan (R.O.C.)) were procured from the Wan Sen Farm (Nantou County Taiwan (R.O.C.)). For the chemical analysis, pullulan standard P-82 kit was procured from Shodex (Tokyo, Japan), the Mushroom and Yeast β-glucan kit was procured from Megazyme (Chicago, IL, USA), Bovine serum albumin (BSA), glucose, meta-hydroxydiphenyl, glucuronic acid, and potassium bromide (KBr) were procured from Sigma-Aldrich (St. Louis, MI, USA). For the antioxidant assay, 2,2-diphenyl-1-picrylhydrazyl (DPPH) was procured from Sigma-Aldrich (St. Louis, MI, USA), 2,2’ -azino-bis-3-ethylbenzthiazoline-6-sulphonic acid (ABTS) was procured from Tokyo Chemical Industry Co., Ltd. (Tokyo, Japan). Dimethyl sulfoxide (DMSO) was procured from J.T. baker (New Jersey, NY, USA), Dulbecco’s Modified Eagle Medium (DMEM), fetal bovine serum (FBS), and Penicillin-Streptomycin-Neomycin (PSN) Antibiotic Mixture were procured from Gibco Laboratories (Grand Island, NY, USA). 3-(4,5-dimethylthiazolyl-2)2,5-diphenyltetrazolium bromide (MTT), 2′,7′-Dichlorofluorescin diacetate and sodium bicarbonate were purchased from Sigma (St. Louis, MI, USA). All other chemicals that were used were of analytical grade.

### 2.2. Preparation of Extract Fraction from F. Velutipes

**Figure 2:**
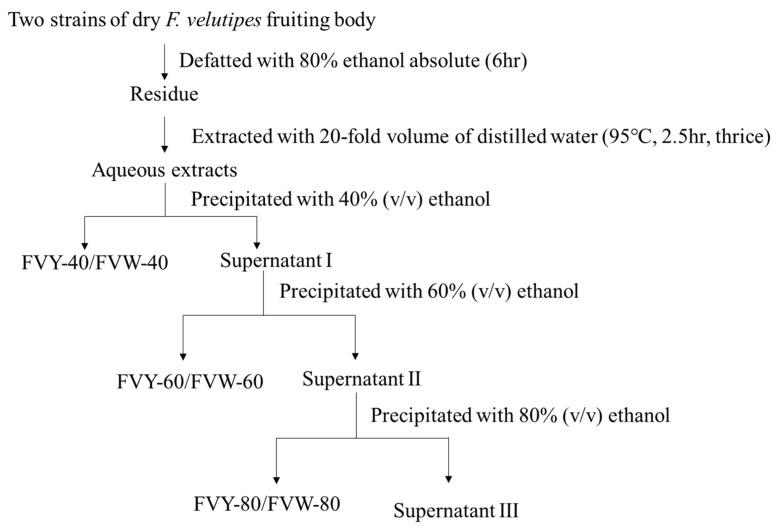
The flow chart of preparation of *F. velutipes* polysaccharides.

The fruiting bodies of two strains of *F. velutipes* were extracted with 80% ethanol to remove the lipids and pigments at 75 °C for 6 h. The residue was then extracted with distilled water while using 65 Hz ultrasonic assisted extraction at 95 °C for 150 min. with the ratio of water to materials of 20 mL/g, and repeated three times. The supernatant was separated from the insoluble residue by centrifugation (2000×*g* for 10 min. at 20 °C). The supernatant was precipitated with 95% ethanol at 4 °C for 24 h to a final concentration of 40% (v/v) and then centrifuged to obtain the precipitate (FVY-40). The supernatant was subsequently precipitated with 95% ethanol (at a final concentration of 60%) and centrifuged to separate the precipitate (FVY-60). The supernatant was subsequently precipitated with 95% ethanol (at a final concentration of 80%) and then centrifuged to separate the precipitate (FVY-80). FVW-40, FVW-60, and FVW-80 were acquired in the same way. The precipitate was collected and lyophilized by vacuum freeze drying. The flow chart of preparation of *F. velutipes* polysaccharides was shown in Figure 2.

### 2.3. Determination of Total Sugar, Uronic Acid, β-Glucan, and Protein Contents

Total sugar content was determined via the phenol-sulfuric acid method while using glucose as a standard [18]. Uronic acid content was determined via the m-hydroxydiphenyl sulfuric acid method using galacturonic acid as the standard [19]. The protein content was determined with the Thermo Scientific™ Micro BCA™ Protein Assay Kit (Waltham, MA, USA), while using bovine serum albumin (BSA) as a standard β-glucan was determined via the Mushroom and Yeast β-glucan Assay using glucose as a standard. All of the samples underwent triple replicate analysis.

### 2.4. Determination of Weight Average Molecular Weight

The weight average molecular weight used high-performance size-exclusion chromatography (HPSEC). FVYs and FVWs were analyzed while using TSK-gel columns (7.8 mm × 300 mm, St. Louis, MI, USA), PW-4000, and PW-3000 connected with TSK-gel PW guard column. The eleuent was 0.3 N NaNO_3_, and the flow rate was 0.5 mL/min. at 70 °C. The linear regression was calibrated with pullulan standards [20].

### 2.5. Monosaccharide Components Analysis

The dried FVYs and FVWs were hydrolyzed by sequential hydrolysis with a combination of methanolysis and trifluoroacetic acid (TFA) to free sugars that are to be determined further by using high-performance anion-exchange chromatography with pulsed amperometric detection (HPAEC-PAD) [21,22]. After alcohol precipitation, 2 mg of dried samples FVYs and FVWs were first dissolved in acetyl chloride and undergoes methanolysation by mixing 1 mL of anhydrous 2 M HCl with absolute methanol under vacuum condition. This reaction was carried out in a closed hydrolytic tube at 80 °C for 16 h. The methanolysis reagent was then evaporated and the methyl glycosides that were produced during methanolysis were further hydrolyzed with 2 M TFA at 120 °C for 4 h. TFA was removed by repeating evaporation under vacuum condition with HPLC-grade distilled water. The free sugars were identified by comparing the retention times with the standard sugars.

### 2.6. FT-IR Spectroscopic Analysis

One to two mg dried sample were mixed with 200 mg of spectroscopic-grade potassium bromide powder and then ground and pressed into pellets for FT-IR measurement. The FT-IR spectra were recorded with Fourier transform infrared (FTIR)-spectrometer at the frequency range of 4000–400 cm^−1^.

### 2.7. Determination of Antioxidant Activities in Vitro

#### 2.7.1. DPPH Radical Scavenging Assay

The measurement of DPPH scavenging activity was modified from previous studies [23,24], while using L-ascorbic acid as the positive control. Subsequently, 100 μL of sample solution at various concentrations were mixed with 100 μL methanol solution containing DPPH radicals (0.2 mM). After vigorously shaking, it was then incubated in darkness at 25 °C for 30 min. The absorbance of the mixture was determined at 517 nm. The scavenging rate of the polysaccharide samples was evaluated by the following Equation:

$$\mathrm{Scavenging}\;\mathrm{ability}\;\left(\%\right)=\left(1-\frac{OD_{sample}}{OD_{control}}\right)\times 100\%$$

where $A_{sample}$ is tested samples ‘ absorbance and $A_{control}$ is the blank’s absorbance (water instead of sample).

#### 2.7.2. ABTS Radical Scavenging Assay

The ABTS scavenging activity was measured referring to the method that was described in the literature with modifications [25,26], while using L-ascorbic acid as the positive control. The stock solution of ABTS•^+^ was diluted to an absorbance of 0.70 ± 0.05 at 734 nm. A 20 μL of sample solution at various concentrations were mixed with 180 μL of ABTS•^+^ solution. The reaction was kept at room temperature for 6 min. in the dark; the absorbance of the mixture was then determined at 734 nm. The scavenging rate of the polysaccharide samples was evaluated by the following Equation:

$$\mathrm{Scavenging}\;\mathrm{rate}\;\left(\%\right)=\left(1-\frac{OD_{sample}}{OD_{control}}\right)\times 100\%$$

where $A_{sample}$ is tested samples ‘ absorbance and $A_{control}$ is the blank’s absorbance (water instead of sample).

### 2.8. Cell Viability Assay

The mouse fibroblast cell line L929 cells (Hsinchu, Taiwan (R.O.C))were cultured in DMEM medium that was supplemented with 10% fetal bovine serum in a humidified atmosphere under 5% CO_2_ at 37 °C. Briefly, 200 μL of L929 cells (8 × 10^3^ cells) were dispensed into 96-well plates. After 24 h, DMEM medium containing different concentrations of FVYs and FVWs add in place of the old medium. After incubation for 24 h, cell viability was determined by adding 100 μL DMEM medium with MTT (5 mg/mL) and then incubated for another 2 h at 37 °C. DMEM medium with MTT (5 mg/mL) was aspirated and the formazan crystals were dissolved by adding 200 μL of DMSO into each well. The absorbance at 570 nm of the formazan solution was recorded while using ELISA reader. The cell viability was calculated using the following formula [9,27]:

$$\mathrm{viability}\;\mathrm{rate}\;\left(\%\right)=\frac{OD_{570\left(treated\;cells\right)}}{OD_{570\left(untreated\;cells\right)}}\times 100\%\;.$$

### 2.9. Intracellular ROS Content

L929 cells (3 × 10^4^ cells) were dispensed into six-well plates overnight, and growth medium was replaced with medium containing different concentrations of FVYs and FVWs. Following incubation for 24 h, the L929 cells were incubated in a medium containing 0.75 mM hydrogen peroxide (H_2_O_2_) for 2 h. Next, the L929 cells were rinsed twice with PBS to remove the remaining ROS and then incubated with 10 µM DCHF-DA for 30 min. in the dark. Images were obtained with a fluorescence microscope with excitation at 488nm and emission at >500 nm. The fluorescent intensities were analyzed while using Image J. (Bethesda, Maryland, USA). All of the images were converted to grayscale images and then inverted and calibrated while using Image J. The mean fluorescence intensities were calculated as mean gray values that were derived from the sum of the gray values of all the pixels in the selection divided by the number of pixels [28,29,30].

### 2.10. Statistical Analysis

For each test, all of the results were expressed as means ± standard deviations. The one-way ANOVA and Duncan’s multiple range tests using SPSS 20 software (New York, USA) was used for the statistical analysis.

## 3. Results and Discussion

### 3.1. Physicochemical Properties of FVYs and FVWs

Table 1 shows the physicochemical properties of FVYs and FVWs. As seen, all of the samples were made up of carbohydrate and protein. The amount of total sugar and uronic acid of FVYs was higher than the corresponding fractions of FVWs. Protein and uronic acid were detected in FVYs and FVWs, which suggests that all of the samples belong to acid glycoprotein compounds [31].

**Figure 3:**
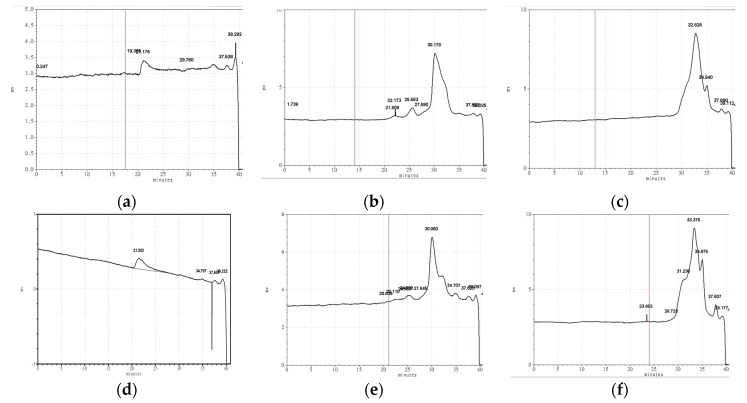
Molecular weight distribution of (**a**) FVY-40, (**b**) FVY-60, (**c**) FVY-80, (**d**) FVW-40, (**e**) FVW-60, and (**f**) FVW-80 determined by high-performance size-exclusion chromatography.

The weight average molecular weight of FVYs and FVWs were determined by high-performance size-exclusion chromatography (HPSEC). The results are shown in Table 2 and Figure 3; the weight average molecular weight of FVY-40, FVY-60, and FVY-80 were 2377.04 kDa, 18.32 kDa, and 3.84 kDa, respectively. The weight average molecular weight of FVW-40, FVW-60, and FVW-80 were 2782.00 kDa, 19.09 kDa, and 2.76 kDa, respectively. The results are consistent with previous studies, namely with low ethanol concentration, the polysaccharides have greater molecular weight, which is caused by the different polarity of the functional groups [32,33]. It was reported that the ethanol concentration was associated to the fractions’ molecular weight [34,35].

### 3.2. Monosaccharide Compositions analysis of FVYs and FVWs

**Figure 4:**
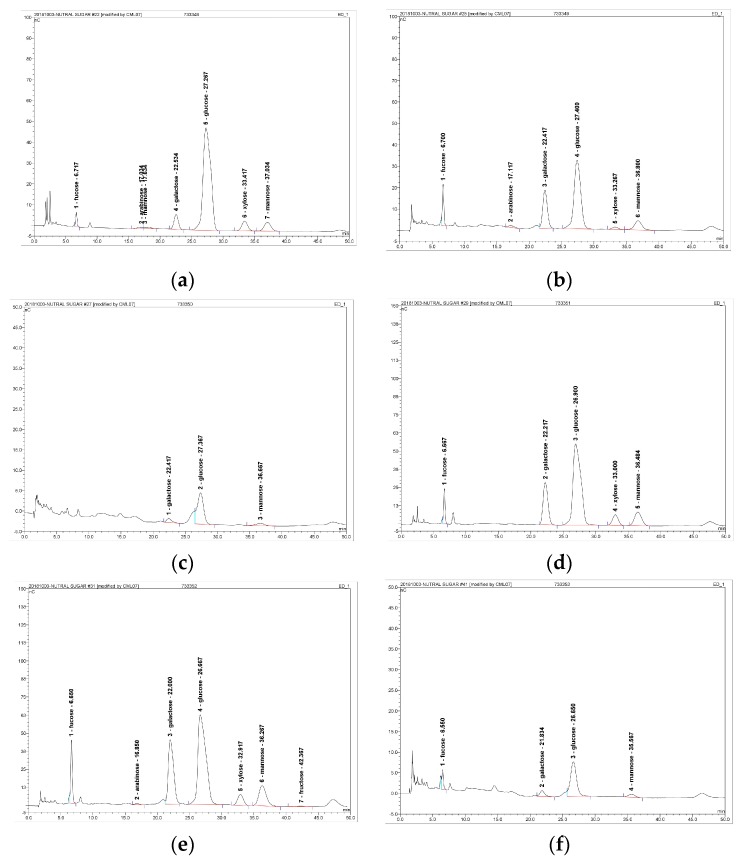
High-performance anion-exchange chromatography with pulsed amperometric detection (HPAEC-PAD) of monosaccharides composition: (**a**) FVY-40, (**b**) FVY-60, (**c**) FVY-80, (**d**) FVW-40, (**e**) FVW-60, and (**f**) FVW-80.

The FVYs and FVWs were hydrolyzed into free sugars under acidic conditions and detected by high performance anion-exchange chromatography with pulsed amperometric detection (HPAEC-PAD) [21]. As shown in Table 3 and Figure 4. The major sugar in both FVYs and FVWs was glucose, followed by galactose and mannose. FVY-40 contained trace amounts of rhamnose, fucose, arabinose, and xylose, while FVY-60 contains trace amounts of fructose and arabinose. FVY-80 contained glucose, mannose, and galactose, and it had the least varieties of sugar units. However, FVW-40 contained small amounts of fructose and xylose, FVW-60 contained small amounts of fructose and arabinose. FVW-80 contained glucose, mannose, fucose, and galactose. Additionally, FVY-40 contained the highest amount of glucose of all of the fractions. It can be proven that fractional precipitation can effectively separate fractions containing different monosaccharide compositions, which is related to their bioactivity [10]. The monosaccharide compositions in FVYs slightly differ from FVWs. FVYs and FVWs showed similar monosaccharide composition to a previous report that demonstrated *F. velutipes* polysaccharide purified by DEAE-52 cellulose column chromatography mainly contained glucose, mannose, and galactose [36].

### 3.3. FTIR Spectra of FVYs and FVWs

**Figure 5:**
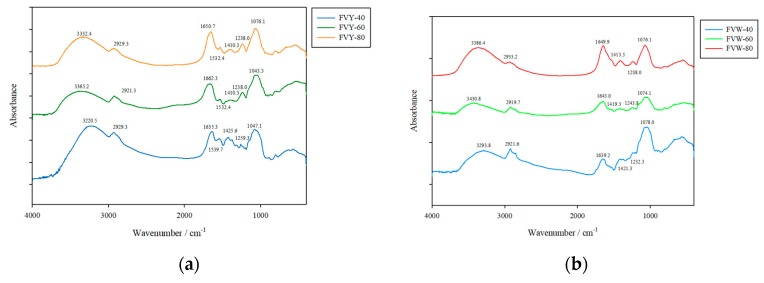
FT-IR spectrum of (**a**) yellow strain and (**b**) white strain polysaccharides at the range of 4000–400 cm^−1^.

FTIR spectroscopy is typically used for the qualitative measurement of organic functional groups, especially O–H, N–H, and C=O. The FT-IR spectra of FVYs and FVWs are shown in Figure 5. The peak around 3450–3200 cm^−1^ was a broad and intense absorption that was attributed to the intermolecular and intramolecular O–H stretching vibration [16]. The band around 2923 cm^−1^ was due to the stretching vibration of C–H, including CH, CH_2_, and CH_3_ in the sugar ring [2]. The relative absorption peak around 1,650 cm^−1^ for N-H bending vibration might be related to the minimal content of protein. The group of bands extending from 1485 cm^−1^ to 1350 cm^−1^ was due to the deforming vibrations of the C–H bond. The absorption band at 1000 cm^−1^ to 1200 cm^−1^ suggests that all of the polysaccharides contained pyranose monomers in the structure. FVYs has similar functional groups to FVWs, according to the FTIR spectra pattern. Copious studies have shown that antioxidant activities are influenced by the –OH group, –NH group, and –COOH group; the –OH and –COOH can donate a hydrogen atom to a radical and convert radicals to non-harmful products, while the –NH_2_ can form –NH_3_^+^ by absorbing hydrogen ions from the solution, and then reacting with the radicals to form non-toxic products, and it can be detected within the FTIR spectra [37], which implies that FVYs and FVWs could possess potential antioxidant capacity.

### 3.4. DPPH and ABTS Radical Scavenging Assay

**Figure 6:**
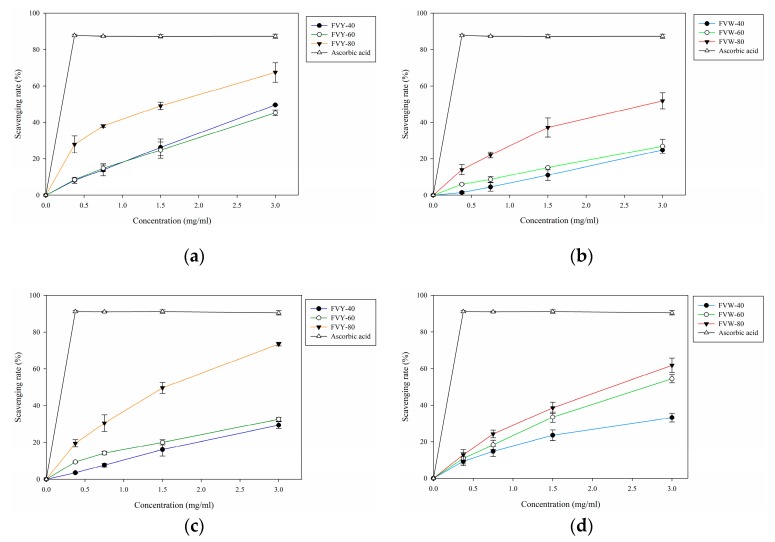
Antioxidant activities of *F. velutipes* polysaccharides (FVYs) and *F. velutipes* polysaccharides (FVWs) *in vitro*. The scavenging rate of (**a**) FVYs and (**b**) FVWs in DPPH radical scavenging assay, and the scavenging rate of (**c**) FVYs and (**d**) FVWs in ABTS radical scavenging assay. The results are presented as means ± standard deviations (*n* = 3).

DPPH is a stable free radical that shows maximum absorption at 517 nm [38], the effect of antioxidant on DPPH radical scavenging was conceived to be due to its proton-donating ability [39,40]. Figure 6a,b showed the results of the experiment. We used fractional precipitation, a simple, rapid, and reproducible method for polysaccharide purification, to separate three fractions. The results indicated that all of the samples showed a concentration-dependent manner. The scavenging rates of FVY-40, FVY-60, and FVY-80 were 49.61%, 45.24%, and 67.46%, respectively, at a concentration of 3 mg/mL, while the scavenging activities of FVW-40, and FVW-60, and FVW-80 were 24.86%, 26.88%, and 51.85%, respectively, at a concentration of 3 mg/mL. IC_50_ of FVY-80, and FVW-80 on DPPH were calculated as 1.57 mg/mL and 2.80 mg/mL, respectively. The scavenging ability of FVY-80 and FVW-80 was similar to *F. velutipes* polysaccharide that was extracted by the enzymatic method (IC_50_ = 2.16 mg/mL) [14], but lower than exopolysaccharide that was extracted from *F. velutipes SF*-*06* (IC_50_ ≤ 1.0 mg/mL) [16].

ABTS assay is widely used in evaluating samples’ total antioxidant power. It employs a specific absorbance (734 nm) and it requires a short reaction time, as an index reflecting the antioxidant capacity of the test sample [40]. The results of ABTS in this study are shown in Figure 6c,d. Concentration-dependent radical scavenging activity was observed. The scavenging rates of FVY-40, FVY-60, and FVY-80 were 29.39%, 33.26%, and 73.43% at a concentration of 3 mg/mL, while the scavenging rates of FVW-40, FVW-60, and FVW-80 were 36.72%, 42.44%, and 61.80% at a concentration of 3 mg/mL, respectively. The best fractions were both found in using a final ethanol concentration of 80%; the IC_50_ of FVY-80 and FVW-80 were 1.52 mg/mL and 2.24 mg/mL, respectively.

The results indicated that a small molecular weight polysaccharide showed a better radical scavenging rate. It was reported that the high-molecular-weight and high-viscosity polysaccharide restricts its mobility and accessibility to the radical [10,41,42]. The weight average molecular weight of FVY-80 and FVW-80 were the smallest among FVYs and FVWs, respectively, explaining why FVY-80 and FVW-80 had the best radical scavenging ability in both DPPH and ABTS radical scavenging assay in FVYs and FVWs, respectively. The weight average molecular weight of *Panax japonicus* C.A. Meyer polysaccharides (PP1, PP2, PP3, PP4, and PP5) that were separated from fractional precipitation were 1.09 × 10^5^, 5.27 × 10^4^, 3.75 × 10^4^, 2.77 × 10^4^, and 4.81 × 10^3^ Da, respectively. Among of them, PP5 possessed the strongest scavenging ability in various antioxidant assays, and IC_50_ of PP5 were <2.5 mg/mL and <1.25 mg/mL in DPPH and ABTS radical scavenging assay, respectively, which is similar to the IC_50_ of FVY-80 [42], indicating molecular weight is one of the factors affecting the antioxidant ability of polysaccharide. Moreover, protein content is one of the factors affecting antioxidant ability of polysaccharide, higher protein content showed better radical scavenging rate. FVY-80 and FVW-80 contained the highest protein content among their fractions, respectively, and showed better antioxidant capacities. That’s because the amino and carboxyl group of protein can donate hydrogen to free radicals and stabilize them [41,42].

According to the antioxidant activities, FVYs exhibited better antioxidant activities than the corresponding FVWs fractions and, among of them, FVY-80 and FVW-80 have the best antioxidant activities in FVYs and FVWs, respectively; the protein contents were 35.85% and 25.95%, which are significantly different. The total sugar contents of FVY-80 and FVW-80 were 37.51% and 36.58%, the β-glucan content was 16.68% and 13.55%, and the uronic acid content was 1.71% and 1.68%, respectively. In general, the total constituents content of FVY-80 is higher than FVW-80, which may be the reason why the antioxidant activity of FVY-80 is better than FVW-80. 

### 3.5. Effect of FVYs and FVWs on Cell Viability and ROS Production of H_2_O_2_-Induced L929 Cells

**Figure 7:**
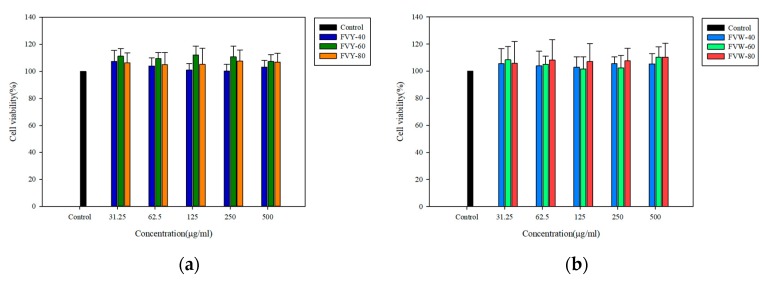
Cell viability was analyzed by 3-(4,5-dimethylthiazolyl-2)2,5 diphenyltetrazolium bromide (MTT) assay. L929 were treated with (**a**) FVYs and (**b**) FVWs for 24 h. Data were expressed as a mean ± standard deviations. (*n* = 4). (*p* < 0.05).

Prior to evaluating the protective capacity of FVYs and FVWs against oxidative stress, L929 was incubated with samples at different concentrations for 24 h. The viability was investigated while using MTT assay to ensure non-cytotoxicity [43]. As shown in Figure 7, after 24 h of cell incubation with various concentrations of samples (31.25, 62.5, 125, 250, and 500 μg/mL), cells viability did not change significantly. The cell viabilities had no significant difference between the control and experimental groups, which indicated that all samples had no cytotoxicity to L929 cells below a concentration of 500 μg/mL.

**Figure 8:**
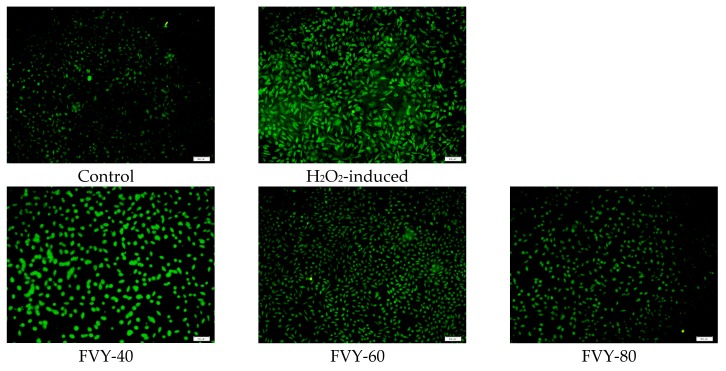

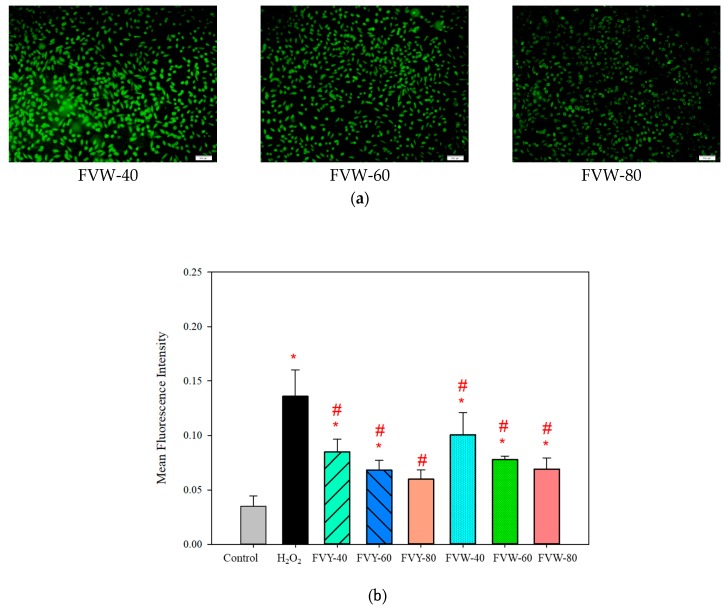
(**a**) Radical scavenging effect of FVYs and FVWs on L929 cells against H_2_O_2_-induced ROS generation by fluorescence microscope. (**b**) All data were expressed as a mean ± standard deviations. (*n* = 3). *p < 0.05 vs. H_2_O_2_-induced group, while #p < 0.05 vs. control group.

It has been reported that H_2_O_2_ injures cells through the production of highly potent oxidizing species, such as •OH, by the Fenton Reaction. ROS, as a major biomarker to assess the oxidative stress level in cell, may not only act as toxic molecules, but also play multiple key roles as signaling molecules regulating various biological processes. Hence, the overproduction of ROS could cause cell apoptosis. [44]. This study hypothesized that FVYs and FVWs probably protected the L929 cells from oxidative stress by modulating the intracellular ROS levels. For verification, this study assessed the intracellular ROS levels in H_2_O_2_-stimulated L929 cells with or without pretreatment with FVYs (500 μg/mL) and FVWs (500 μg/mL) while using DCFH-DA assay. The results are shown in Figure 8a, H_2_O_2_ did significantly increase the fluorescence intensity in H_2_O_2_-stimulated L929 cells, as compared with the control group, and FVY-80 and FVW-80 greatly reduced the fluorescence intensity in the H_2_O_2_-stimulated L929 cells. As shown in Figure 8b, the mean fluorescence intensity of L929 cells treatment with H_2_O_2_ was 0.1358 and that of H_2_O_2_-stimulated L929 cells pretreatment with FVY-40, FVY-60, FVY-80, FVW-40, FVW-60, and FVW-80 were 0.0849, 0.0683, 0.0598, 0.1007, 0.0776, and 0.0692, respectively. The mean fluorescence intensity of H_2_O_2_-stimulated L929 cells pretreatment with FVY-40, FVY-60, FVY-80, FVW-40, FVW-60, and FVW-80 were decreased by 37.48%, 49.71%, 55.96%, 25.85%, 42.86%, and 49.04%, respectively, when compared with the control group. FVY-80 showed the greatest ROS scavenging activity among FVYs and FVWs, and it provided a protective effect against oxidative stress in H_2_O_2_-stimulated L929 cells. It has been reported that *F. velutipes* polysaccharide has the superoxide anion radical scavenging ability and hydroxyl radicals scavenging ability [45,46]. Exopolysaccharide extracted from the culture of *F.velutipes* SF-06 increased the catalase (CAT) activity in the heart, liver, kidney, and blood [16] and *F.velutipes* SF-08 mycelia polysaccharide significantly enhanced the activities of antioxidant enzymes, such as superoxide dismutase (SOD) and glutathione peroxidase (GSH-Px) activity, in aging mice [46], which suggested that FVYs and FVWs might increase the antioxidant enzymes against oxidative stress in H_2_O_2_-stimulated L929 cells.

## 4. Conclusions

In our work, we looked into the physicochemical properties, antioxidant activity, and biological activity of the FVYs and FVWs. According to the results, FTIR spectra and HPAEC-PAD showed that FVYs and FVWs contain –OH group, –NH group, and –COOH group, belong to acid hetero polysaccharide, and have a different molar ratio of monosaccharide composition. In terms of antioxidant activities, the FVYs showed better antioxidant properties than the corresponding FVWs, and we found that we could obtain better antioxidant fractions through fractional precipitation; FVY-80 was the best antioxidant fraction among all fractions, which might be owed to their physicochemical properties. Furthermore, FVYs and FVWs did not show cytotoxicity toward L929 cell under a concentration of 500 μg/mL and FVY-80 showed the best protection against oxidative stress, and it effectively decreased the ROS production. However, the mechanism of how FVYs and FVWs decrease the oxidative stress in H_2_O_2_-stimulated L929 cells and other biological functions of FVYs, such as anti-aging and improvement wound healing, are under on-going research. This study shows that yellow strain *F. velutipes* is not only palatable food, but is also a potential source of an antioxidant cosmetic ingredient, providing a certain level of protection against oxidative stress.

## Figures and Tables

**Table 1:** Chemical composition of yellow strain and white strain polysaccharides. The results are presented as means ± standard deviations (*n* = 3).

Sample	Total Sugar Content (%)	Uronic Acid Content (%)	β-Glucan Content (%)	Protein Content (%)
FVY-40	83.13 ± 1.29 ^Aa^	2.89 ± 0.36 ^Aa^	44.56 ± 1.77 ^Aa^	15.52 ± 3.14 ^Cb^
FVY-60	75.94 ± 5.03 ^ABa^	2.10 ± 017 ^Bb^	18.45 ± 0.49 ^Cb^	17.15 ± 3.45 ^Cb^
FVY-80	41.10 ± 2.09 ^Db^	1.71 ± 0.31 ^Bb^	16.68 ± 1.62 ^Cb^	35.85 ± 2.91 ^Aa^
FVW-40	77.99 ± 0.87 ^ABd^	2.02 ± 0.05 ^Bd^	44.18 ± 0.27 ^Ad^	14.16 ± 1.38 ^Ce^
FVW-60	60.54 ± 3.64 ^Ce^	1.81 ± 0.06 ^Be^	26.73 ± 2.11 ^Be^	23.60 ± 0.99 ^Bd^
FVW-80	40.87 ± 5.21 ^Df^	1.68 ± 0.14 ^Be^	13.55 ± 0.34 ^Df^	25.95 ± 0.25 ^Bd^

^A–D^ Values in the same column not sharing a common superscript letter are significantly different at *p* < 0.05 between two *F. velutipes* polysaccharide. ^a–c^ Values in the same column not sharing a common superscript letter are significantly different at *p* < 0.05 within the yellow strain polysaccharide. ^d–f^ Values in the same column not sharing a common superscript letter are significantly different at *p* < 0.05 within the white strain polysaccharide.

**Table 2:** The weight average molecular weight of (**a**) yellow strain and (**b**) white strain polysaccharides by fractional precipitation.

	40%	60%	80%
Yellow strain	2377.04 kDa	18.32 kDa	3.84 kDa
White strain	2782.00 kDa	19.09 kDa	2.76 kDa

**Table 3:** Monosaccharide composition of yellow strain and white strain polysaccharides.

Monosaccharide Composition		Yellow Strain			White Strain		
		40%	60%	80%	40%	60%	80%
	Glucose	87.90	54.50	75.00	66.49	58.02	64.22
	Fructose	-	-	-	-	0.25	-
Molar	Mannose	5.55	11.76	14.29	11.89	14.80	9.88
Percentage	Rhamnose	0.81	-	-	-	-	-
(%)	Fucose	1.36	11.90	-	3.73	7.24	13.55
	Galactose	2.59	21.03	10.71	15.37	18.79	12.35
	Arabinose	0.29	0.82	-	-	0.10	-
	Xylose	1.49	-	-	2.53	0.81	-

-, Trace or undetectable.
